# Impact of community treatment orders on inpatient bed usage in assertive outreach team

**DOI:** 10.1192/bjo.2021.625

**Published:** 2021-06-18

**Authors:** Mohammed Al-Uzri, Zena Harvey, Fabida Noushad, Chinyere Iheonu, Mohammed Abbas

**Affiliations:** 1 Leicestershire Partnership Trust; 2 Nottinghamshire Healthcare NHS Foundation Trust

## Abstract

**Aims:**

To examine the impact of using Communty Treatment Orders (CTO) of the Mental Health Act on use of inpatient care in Assertive Outreach team.

**Background:**

Currently there is little evidence of the efficacy of community treatment orders (CTOs), and in particular with patients who use the Assertive Outreach service. One large randomised controlled study found no impact on use of inpatient care while a naturalistc study found significant impact.

**Method:**

Our primary outcome was the number of admissions with and without a CTO comparing each patient with themselves before CTO and under CTO(“mirror-image”). Our secondary outcomes were the number of bed days, and the percentage of missed community visits post-discharge. We also looked at the potential cost savings of a reduction in inpatient bed usage.

**Result:**

All the 63 patients studied over period of 6 years had a severe and enduring mental illness. The use of a CTO was linked to a significant reduction in the number of admissions (mean difference = 0.89, 95% CI = 0.53–1.25, P < 0.0001) and bed days (mean difference = 158.65, 95% CI = 102.21–215.09, P < 0.0001) There was no significant difference in the percentage of missed community visits post-discharge. Looking at the costs, an average cost for an inpatient Assertive Outreach bed per day in the local Trust was £250, and there were 8145 bed days saved in total, making a potential saving of just over £2million, during the study period.

**Conclusion:**

This study suggests that the implementation of CTOs using clinical judgment and knowledge of patients can significantly reduce the bed usage of Assertive Outreach patients. The financial implications of CTOs need to be reviewed further, but this study does suggest that the implementation of CTOs is a cost-effective intervention and is economically advantageous to the local Trust.

